# Clinical usefulness of the teller acuity cards test in preliterate children and its correlation with optotype test: A retrospective study

**DOI:** 10.1371/journal.pone.0235290

**Published:** 2020-06-29

**Authors:** Hye Jun Joo, Ho Chul Yi, Dong Gyu Choi

**Affiliations:** Department of Ophthalmology, Hallym University College of Medicine, Kangnam Sacred Heart Hospital, Seoul, Korea; Faculty of Medicine, Cairo University, EGYPT

## Abstract

This study evaluated the reproducibility of the Teller Acuity Cards (TAC) test, its correlation with the optotype test, and its usefulness for detecting amblyopia in preliterate children. We retrospectively reviewed the medical records of 64 children who had undergone the TAC test more than once and were later followed up with the optotype test. The mean corrected visual acuities (logMAR) of the first and last TAC tests were 0.86 (mean 19.9 months) and 0.69 (27.7 months), respectively. The first optotype acuity was 0.18 (33.7 months). The first TAC acuity result was positively correlated with the age of the child, but it was not statistically significant (r = −0.077, p > 0.05). The first and last TAC test acuities were significantly correlated (r = 0.382, p < 0.01). There was a significant but small correlation between the final TAC and the first optotype acuities (r = 0.193, p < 0.05). Interocular differences in visual acuity were significantly correlated between the last TAC and first optotype tests (r = 0.395, p < 0.05). TAC acuity might be a valid predictor of optotype acuity later on although it was underestimated compared to that in the optotype test. The TAC test can be used to detect unilateral amblyopia in preliterate children.

## Introduction

The Teller Acuity Cards (TAC) test is one of the preferential-looking (PL) procedures and has been widely used in laboratory and clinical settings for the assessment of grating acuity of nonverbal children and infants [1, 2]. Atkinson and Braddick [3, 4] analyzed acuity development in a large sample of infants having a first-degree relative with a history of amblyopia and/or strabismus. They reported that the initial Forced-choice Preferential Looking (FPL) estimates obtained between 8 and 11 weeks of age did not correlate significantly with those obtained between 6 and 9 months of age. Courage and Adams [5] also reported poor prediction of acuity results over short intervals, at least within the first year, in healthy full-term infants.

In contrast, significant correlations between earlier and later PL results were reported in some studies. Maurer et al. [6] compared FPL acuity results obtained at 12, 18, 24, 30, and 36 months of age with recognition letter acuity estimates collected after four years old in 35 patients with congenital cataract and significant predictive correlations were observed between FPL acuity results and recognition acuity later in life. Birch et al. [7] also reported that FPL estimates at 24, 36, and 48 months of age were correlated with recognition acuity at >60 months for the aphakic eye in a 14 children sample. However, the sample sizes of these studies were relatively small.

The aim of this study was to evaluate the reproducibility of the results of the TAC test, its correlation with the optotype test, and its usefulness as an early screening tool for detecting amblyopia in nonverbal children.

## Materials and methods

### Study design and subjects

We retrospectively reviewed medical records of children who had visited the pediatric ophthalmology clinic of Kangnam Sacred Heart Hospital, Hallym University, and had undergone the TAC test more than once during the preliterate period (ages <3 years old) and later, once they became literate, tested in the optotype visual acuity measurement. Patients with ocular structural abnormalities including retinopathy of prematurity, retinal or corneal disease, or a history of ocular operations including strabismus surgery, were excluded from the initial medical records review. However, patients with refractive errors, strabismus or epiblepharon were included. Cycloplegic refraction with 1% cyclopentolate and 1% tropicamide and a slit-lamp biomicroscopic and fundus examination were performed in all patients. All deviations were measured with the alternate prism cover test at near and distance points, 0.3 m and 6 m, respectively, or the modified Krimsky method in uncooperative children (with spectacle correction based on cycloplegic refraction, if necessary). This study’s protocol adhered to the Declaration of Helsinki and was approved by the Institutional Review Board of Hallym University Medical Center (approval no. 2019-09-006). Informed consent was waived owing to the retrospective nature of the study.

### Visual acuity measurement procedures: Grating visual acuity (TAC) & optotype visual acuity

Grating visual acuity was measured using TAC assessment (Vistech Consultants, Inc., Dayton, OH, USA). The set of cards consisted of 17 gray cardboard cards with square-wave gratings ranging in spatial frequency from 0.23 to 38 cycles/cm and one-half an octave steps displayed opposite a blank gray card bearing no stripes. The tester viewed the children’s face through a 4-mm peephole in each card during testing. Under constant illumination conditions (luminance 130–150 cd/m^2^), the test was conducted by one well-trained technician with cards placed 55 cm away from the children’s eye, with optical correction when necessary.

The children were usually more interested in the striped side than the non-striped side and thus moved their eye or head toward the striped side [8]. The examiner judged the children’s reaction by presenting a start card without knowing whether the stripes were on the left or on the right side. The examiner then rotated the card 180° and presented it for a second time. If the children looked in the opposite direction, the examiner checked the location of the stripes. If a child’s line of sight was the same as the striped side, the examiner chose a card with a spatial frequency one octave higher. The children’s’ visual acuity was determined by the card with the thinnest stripes for which the subject managed two correct answers. Results were obtained in cycles/cm and expressed as logMAR values.

Optotype visual acuity was measured using Hahn's visual acuity chart, which is one of Snellen visual acuity charts developed in Korea. It consists of three parts measuring visual acuity ranging from 20/200 to 20/10 using numbers, pictures, and Landolt rings. Acuity was measured using the best optical correction at 6-m distance.

### Main outcome measures

First, the relationship between age and visual acuity in the TAC test was evaluated. Second, test-retest reproducibility of the TAC test was evaluated by comparing the first and last TAC test results. Third, the last TAC test acuity was compared with the results of the first optotype acuity test to estimate the predictive validity of the TAC test. Finally, the interocular difference in visual acuity in the TAC test was compared with that in the optotype acuity test to assess the usefulness of the TAC test in detecting unilateral amblyopia. This measurement was obtained by subtracting the visual acuity (logMAR) of the left eye from that of the right eye.

### Statistical analyses

Statistical analyses were performed using SPSS for Windows, version 24.0.K (IBM Corp., Armonk, NY, USA). Pearson correlation analysis was performed to compare the results. A *p*-value <0.05 was considered statistically significant.

## Results

Demographic data of the subjects are presented in Table 1. In total, 128 eyes of 64 children (26 males and 38 females) were included in this study. When the TAC visual acuity was assessed for the first time, the mean age was 19.86 ± 5.67 months (range: 8 to 34 months), and when assessed for the last time, the mean age was 27.67 ± 5.57 months (range: 14 to 40 months). The average interval between the first and last TAC tests in the same subject was 7 months. The mean age when the optotype visual acuity was assessed for the first time was 33.67 ± 7.56 months (range: 23 to 61 months). The interval between the last TAC test and the first optotype test was approximately 6 months (range: 1 to 47 months). In the first TAC test, the mean corrected visual acuity was logMAR 0.86 ± 0.29 (range: 0.37 to 1.85), and in the last TAC test, it was logMAR 0.69 ± 0.23 (range: 0.20 ~ 1.37). The mean corrected visual acuity in the optotype test was logMAR 0.18 (range: 0 ~ 0.699) (Table 2).

**Table 1:** Demographic data of enrolled subjects (N = 64 children, 128 eyes).

Sex	
Male/female	26 (40.6)/38 (59.4)
Exotropia/exophoria	15 (23.4)
Esotropia/esophoria	14 (21.9)
Epiblepharon	11 (17.2)
Refractive errors (N = 128 eyes)	
Hyperopia^†^	54 (42.2) 2.78 ± 2.16 [+8.38 to +0.75]
Myopia^‡^	22(17.2) −2.62 ± 3.38 [−0.63 to −12.75]

Values are presented as n (%) or mean ± standard deviation [range].

^†^ >+0.5 diopter by spherical equivalent.

^‡^<−0.5 diopter by spherical equivalent.

**Table 2:** Mean corrected visual acuities and mean ages when assessed.

	Acuity (logMAR)	Age (months)
Teller Acuity Cards test		
First examination	0.86 ± 0.29 [0.37–1.85]	19.86 ± 5.67 [8–34]
Last examination	0.69 ± 0.23 [0.20–1.37]	27.67 ± 5.57 [14–40]
First optotype test	0.18 ± 0.15 [0–0.699]	33.67 ± 7.56 [23–61]

Values are presented as mean ± standard deviation [range].

**Fig 1:**
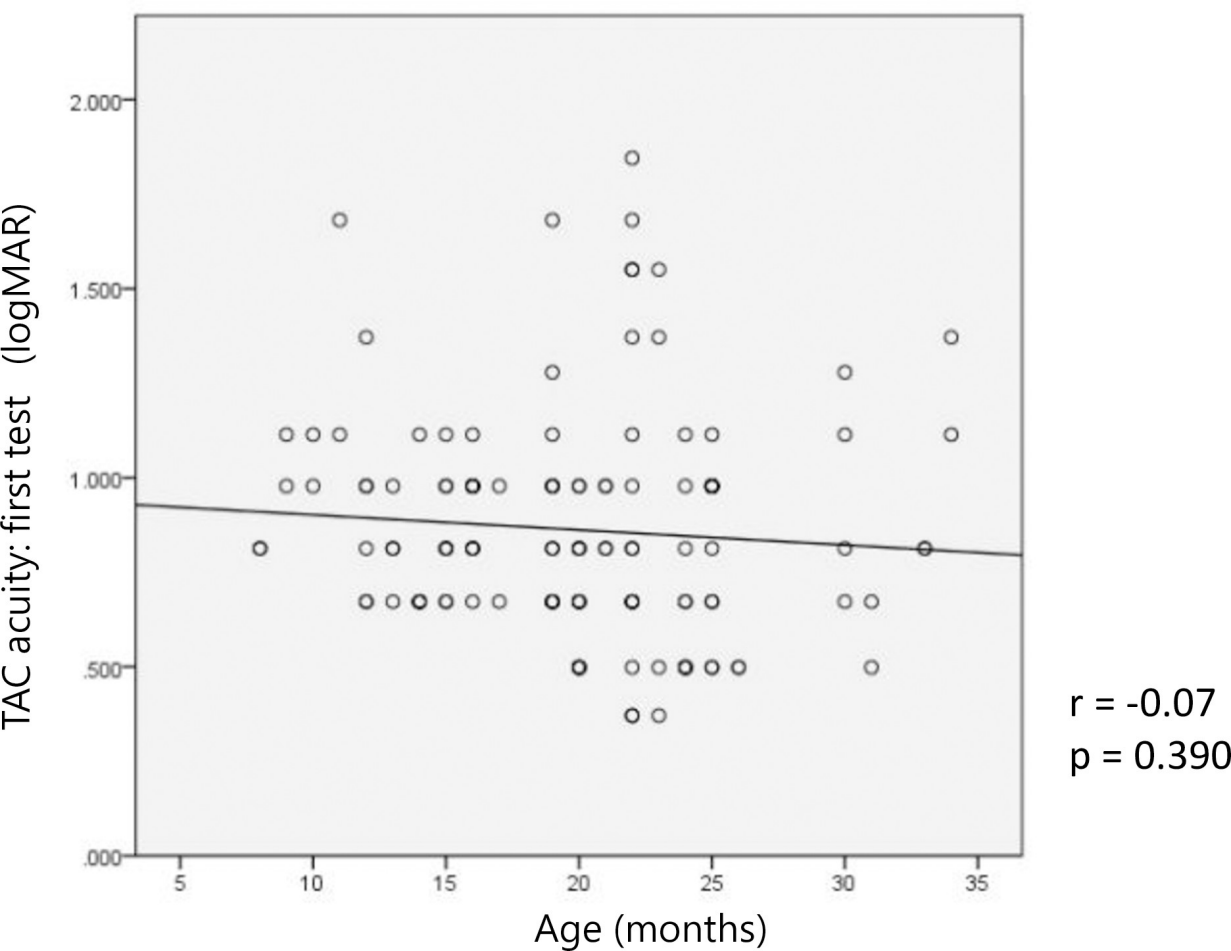
Correlation between the result of the first Teller Acuity Cards (TAC) test and age.

Fig 1 shows the relationship between age and the first visual acuity results using TAC. The TAC visual acuity tended to improve slightly with age, but it was not statistically significant (r = −0.077, p = 0.390).

**Fig 2:**
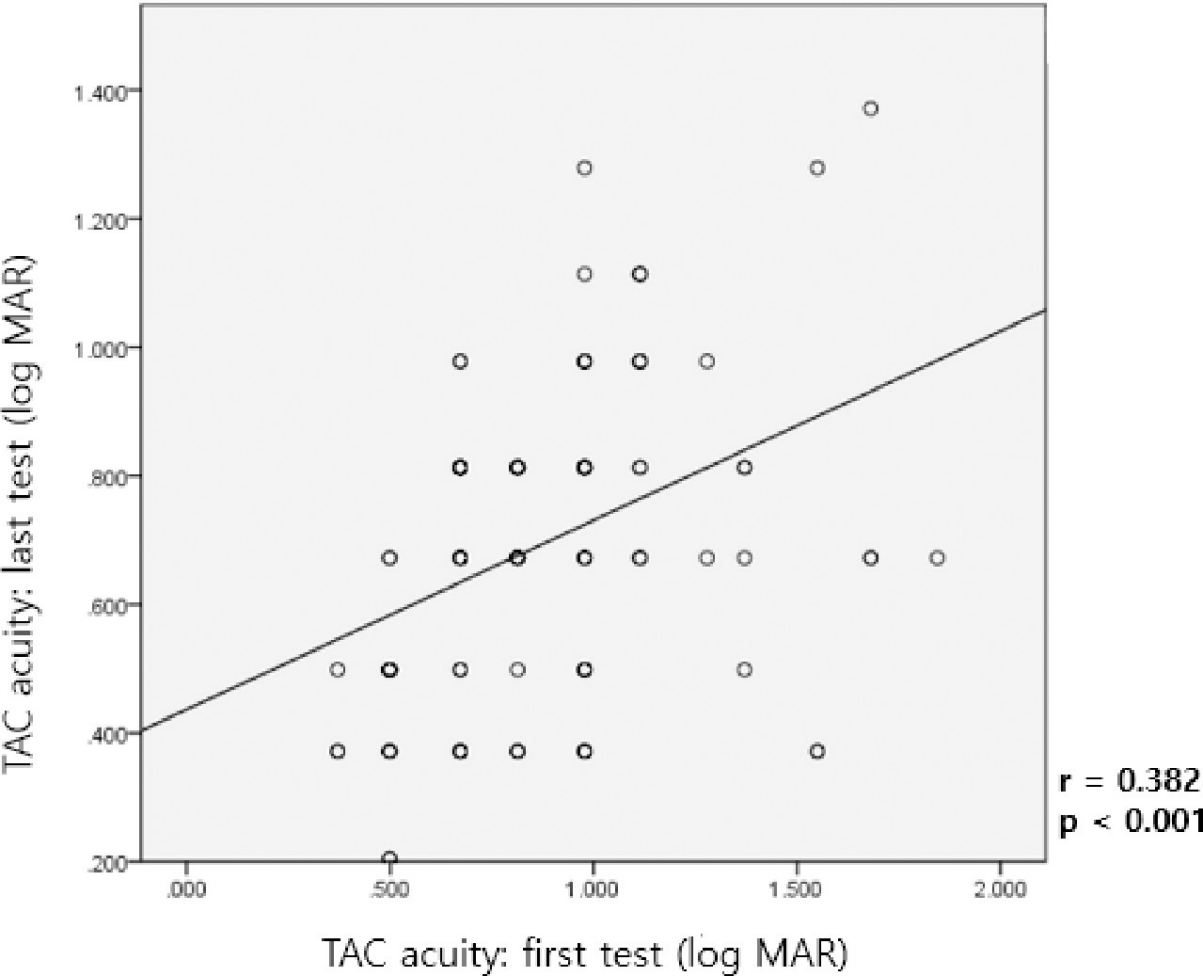
Correlation between the first and last visual acuities in the Teller Acuity Cards (TAC) test.

Fig 2 shows moderate positive correlation between the first and last visual acuities using the TAC test in the same subject, and the result was statistically significant (r = 0.382, p < 0.001).

**Fig 3:**
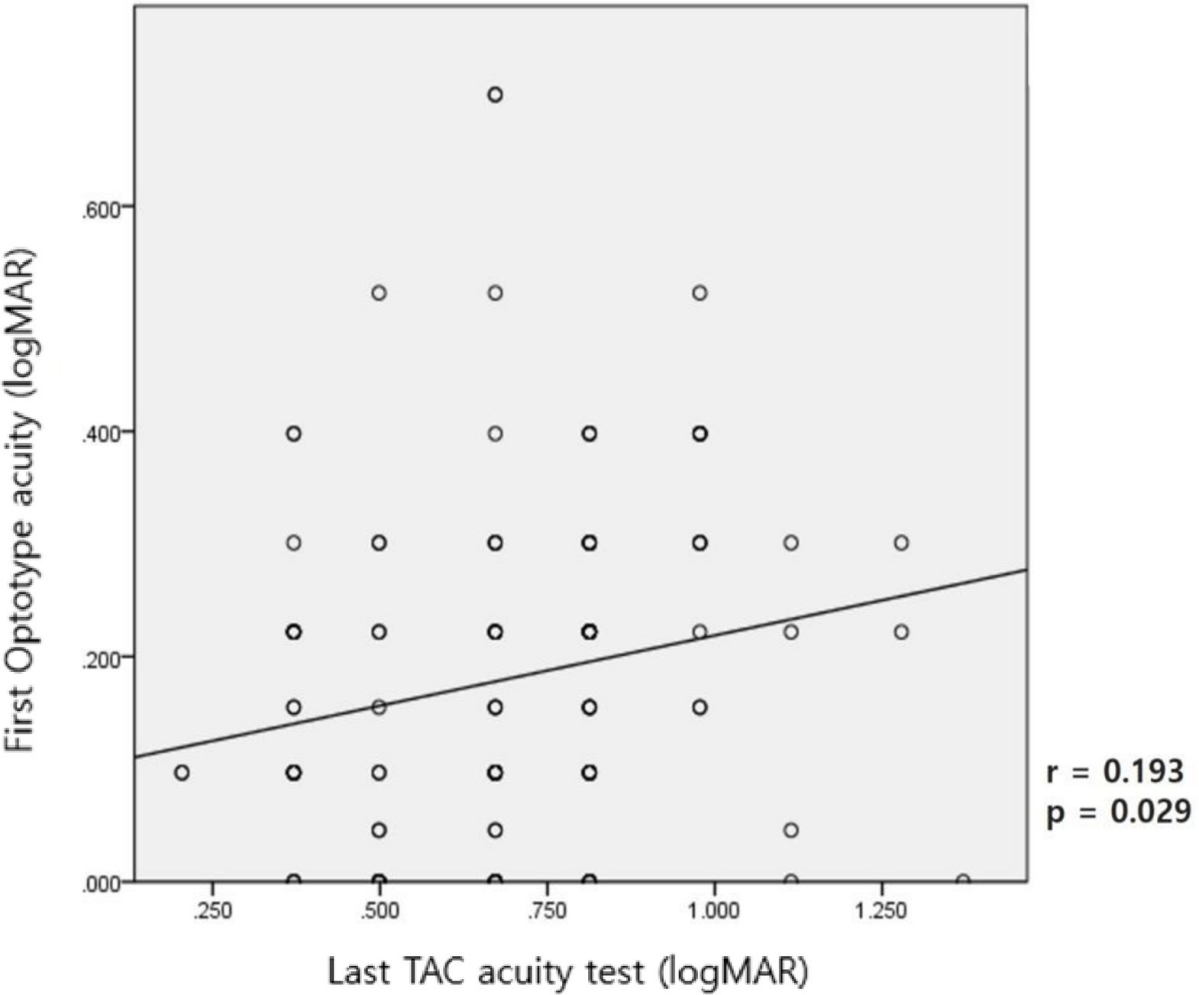
Positive but weak correlation between the last Teller Acuity Cards (TAC) test and the first optotype acuity test.

Fig 3 shows positive but weak correlation between the last TAC test and the first optotype test, with an interval of approximately 6 months in the same subjects, and the results were statistically significant. The TAC test tended to underestimate visual acuity compared with the optotype test.

**Fig 4:**
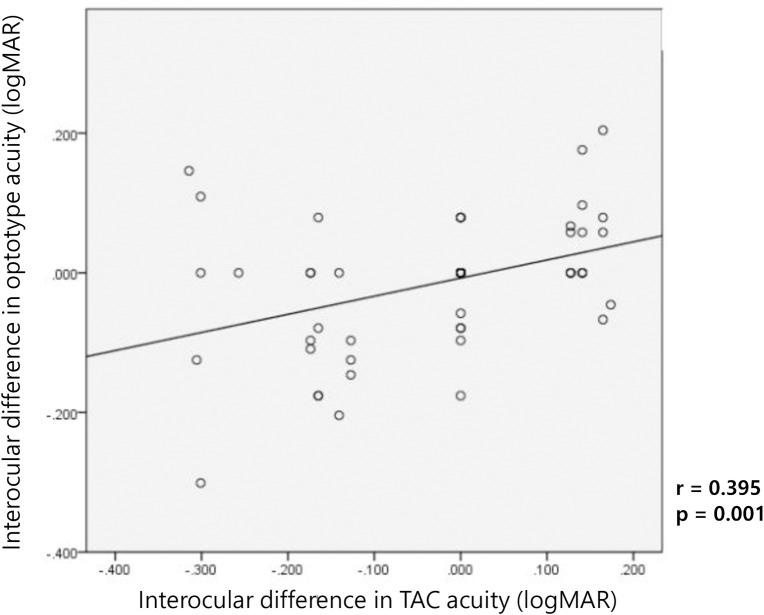
Significant correlation between interocular differences in visual acuity in the TAC and optotype tests.

Fig 4 shows the interocular differences in visual acuity between the TAC and optotype tests, and there was strong positive correlation between both examinations (r = 0.395, p = 0.001), and the result was statistically significant. The TAC test was able to detect monocular amblyopia in young children in whom the optotype test was not possible.

## Discussion

Preferential-looking procedures are widely used psychophysiological methods for assessing visual acuity in infants or young children who cannot verbally express their opinions. However, it is difficult to directly compare visual acuity converted to stripe spacing (PL procedures) with the standard optotype visual acuity test, and there have been controversies concerning the reliability of the TAC test. In addition, studies have been attempted to investigate test-retest reproducibility, reliability between different testers, and predictability of testing in the TAC test, through comparison of visual acuity at the same time point or after growth in which the results were variable and controversial [2, 5, 9, 10]. Courage and Adams [5] reported a longitudinal study of 27 full-term infants who were tested with the TAC test at least twice (mean test-retest interval, 5 months) within the first year of life. They generally concluded that the initial estimates of visual acuity did not predict future estimates. These results were difficult to trust because of the difference in visual acuity development due to the growth of infants and young children and the mood-state at the time of testing. Mash and Dobson suggested that the normal range of the TAC test would show rapid improvement in visual acuity with growth in babies <12 months old [10].

However, in a study of children >12 months old, the TAC test showed relatively high long-term reproducibility and a significant correlation with the optotype test. Birch and Spencer [11] compared the TAC test at the age of 12 and 18 months in 24 children with retinopathy of prematurity and found a high correlation. Similar results were also found in a study of children with aphakia >24 months old, which showed a significant correlation with long-term reproducibility between test-retest and postgrowth optotype visual acuity test [7]. Another study with 45 normal subjects showed that in children with normal visual acuity during infancy and adolescence, normal vision could be predicted following growth and development; however, in children with worse mean visual acuity, the predictability was lower [9]. Mash and Dobson [10] conducted a larger prospective long-term study of 129 newborns. From the follow-up period of 4–48 months, the TAC test showed a statistically significant correlation between the test and the retest, and there was a relatively high correlation between the TAC test at each time point and the optotype test at 48 months.

In this study, we found a statistically significant but moderate positive correlation between the test and retest scores (with an average interval of 6 months between the first TAC and last TAC tests (Fig 2)). These results suggest that the TAC test has relative reproducibility in infants and young children who clinically needed the TAC test. There was no significant difference in the reproducibility of the TAC test according to visual acuity. The reproducibility of the TAC test was comparable regardless of the degree of visual acuity.

The grating visual acuity test using the TAC evaluates visual acuity with a striped card, unlike the optotype visual acuity test, which uses letters or pictures in adults or older children. Thus, it would be somewhat unreasonable to compare the results of these two tests directly. According to Kushner et al. [12], 69 literate children with amblyopia who could be examined with the optotype test had better visual acuity on the TAC test than on the optotype test. Some Korean studies also showed that the TAC test tended to overestimate visual acuity, especially in amblyopic children [13, 14]. This might be because the near visual acuity would be better than the distant visual acuity in amblyopic children; thus, the TAC test, which measures near visual acuity, could somewhat overestimate overall visual acuity [12–14].

However, this study revealed that the TAC test tended to underestimate visual acuity compared to the optotype test. This discrepancy might be because most previous studies comparing the results of the grating and the optotype visual acuity tests, was done at the same time, but in this study, the average interval between the two tests was approximately six months. Visual acuity tends to improve as children grow up regardless of the assessment methods. Similar to our study, other studies in children who were unable to undergo optotype visual acuity test showed that the TAC test could underestimate visual acuity [2, 15].

In this study, the interocular difference in visual acuity using the grating and the optotype tests showed a strong positive correlation between the two tests (r = 0.395, p = 0.001) compared with the analysis of the absolute visual acuity values of the two tests (r = 0.193, p = 0.029). This suggests that the grating acuity test using TAC might be useful in detecting unilateral amblyopia.

A limitation of this study in relation to its retrospective design was the variability in the age and follow-up interval when the TAC test or optotype tests were performed. Furthermore, some patients had strabismus or refractive errors, which would somewhat affect the visual acuity results. As a matter of fact, in the retrospective setting, it was not easy to obtain TAC test measurements more than once in the preliterate period and optotype visual acuity later on in children who were completely normal infants since they do not usually require regular eye clinic visits. However, on the other hand, because we performed this study to evaluate the usefulness of the TAC test for detecting amblyopia in nonverbal children, as well as assessing its reproducibility and correlation with optotype test, it would be better to not exclude patients with refractive error or strabismus to detect anisometropic or strabismic amblyopia. A prospective study evaluating reproducibility and predictability using a larger number of similarly aged subjects undergoing the TAC and optotype tests is needed to overcome these limitations.

## Conclusion

The TAC test can be used as a relatively reliable method for estimating visual acuity in preliterate children. TAC acuity assessment might be a valid predictor of optotype-measured acuities later on in literate children, although the TAC test underestimates visual acuity compared with optotype test. The TAC test can be used to detect unilateral amblyopia, and thus patients could benefit from amblyopia management in preliterate children.
